# Retraction Note: High throughput computations of the effective removal of liquified gases by novel perchlorate hybrid material

**DOI:** 10.1038/s41598-025-23572-0

**Published:** 2025-10-14

**Authors:** Tomsmith O. Unimuke, Hitler Louis, Onyinye J. Ikenyirimba, Gideon E. Mathias, Adedapo S. Adeyinka, Chérif Ben Nasr

**Affiliations:** 1 Computational and Bio-Simulation Research Group, University of Calabar, P.M.B 1115, Calabar, Nigeria; 2 Department of Pure and Applied Chemistry, University of Calabar, P.M.B 1115, Calabar, Nigeria; 3 Department of Chemical Sciences, Research Centre for Synthesis and Catalysis, University of Johannesburg, Johannesburg, 2006 South Africa; 4 Laboratoire de Chimie des Matériaux, Faculté des Sciences de Bizerte, Université de Carthage, 7021 Zarzouna, Tunisie

Retraction of: *Scientific Reports* 10.1038/s41598-023-38091-z, published online 05 July 2023

The Editors have retracted this Article.

Following publication, concerns were raised regarding inaccurate values presented in Tables 1, 2, 5 and 6. The Authors stated that they have made errors in this Article, including the conversion of units from eV to kcal/mol, thermodynamic calculations, calculation of the free energy correction term, and use of inaccurate units of the Boltzmann constant during the calculation of recovery time. Additionally, the Authors stated that most of the calculations in the Article are based on energies obtained using the M062x-D3/6-311++G(2df, 2pd) and DSD-PBEP86/aug-cc-pVDZ, and the generalisation that all calculations were done at the M062X-D3BJ/aug-ccpVTZ is not true.

The Editors therefore no longer have confidence in the presented results.

Tomsmith O. Unimuke, Onyinye J. Ikenyirimba, and Adedapo S. Adeyinka agree to the retraction and its wording. Hitler Louis, Gideon E. Mathias, and Chérif Ben Nasr have not responded to the Editors about this retraction.

